# Arrested pneumatization of sinus sphenoid, revealed by hypo-acusis: A case report

**DOI:** 10.1016/j.amsu.2021.102939

**Published:** 2021-10-13

**Authors:** M. Siradji Harouna, Fadoul Achta, Fatiha Aghrib, Tressia Boussa, Hasna Belgadir, Naima Elbenna

**Affiliations:** a Faculty of Medicine and Pharmacy, Hassan II University of Casablanca, B.P 5696, Casablanca, Morocco; b Radiology Department, CHU Ibn Rochd, B.P 2698, Casablanca, Morocco

**Keywords:** Arrested pneumatisation, Imaging criterias, Hypo-acusis

## Abstract

The sphenoid bone is a complex structure in terms of its embryological origin. At birth, the sphenoid sinus is not pneumatised. Pneumatisation begins at around 4 months of age until the age of 12–14 years.

If this process is absent or interrupted for reasons that are often unknown, it is called arrested pneumatisation.

This report describes the case of a 15 year old patient, who consulted an ENT specialist for chronic headaches and hearing loss on the left side. Clinical ENT examination (including otoscopy) is normal. Tonal audiometry revealed a sensorineural hearing loss in the left ear. A CT scan of the petrous bone was normal but revealed a hypodense lesion in the left sphenoid bone. Lipoma was suggested. A brain MRI was performed in a clinic to better characterize the lesion. MR images showed a well-defined lesion with fatty content. The diagnosis was nasosinus fibrous dysplasia.

In view of the diagnostic discrepancy, the patient was referred to our department for a specialist opinion. An additional brain scan revealed a non-eroded, non-expansive fatty density lesion with well-defined internal curvilinear calcification in the left sphenoid sinus location. Our final diagnosis was arrested pneumatisation.

Most patients with arrested pneumatization of the skull base are asymptomatic. Sometimes it may be revealed by nonspecific signs and be confused with severe skull base disease, especially if the radiologist is not familiar with its existence or its typical features.

## Highlights

• Arrested pneumatization is the absence or incomplete pneumatization of the sphenoidal sinus.
• It is often discovered incidentally but can be revealed by non-specific symptoms such as headache, hearing loss.
• It is a normal variant, recognition of its typical features on imaging is important for the radiologist .
• No additional treatment or check-up is required.

## 1 Introduction

The sphenoid bone represents a complex structure in terms of anatomy and embryology. Indeed, it is formed by the fusion of different primordia whose embryonic origins are different the orbitosphenoid and the basi-post-sphenoid derive from the cephalic mesoderm whereas the alisphenoid and the basi-pre-sphenoid are from neural crest cell origin. The sphenoid bone has been linked with several developmental diseases. Sphenoid sinuses are two pneumatised structures located in the body of the sphenoid bone [[1], [2]].

Classically, the maxillary, sphenoid and frontal sinuses develop by expansion of certain ethmoidal cells in the neighbouring bones of mesenchymal origin that surround the ethmoid the ethmoid, namely the maxillary, frontal and sphenoidal bones [3].

The normal process of pneumatization of the skull base and paranasal sinuses starts in utero and develops through young adulthood. It is known that the red bone marrow is replaced by the fatty marrow prior to the normal pneumatization process of the paranasal sinuses, including the sphenoid bones. The process of marrow conversion occurs before epithelialization and the formation of the respiratory mucosa in the aerated sinus. In the sphenoid, the fatty conversion usually begins at around four months of age, and ends at 10–14 years of age. The fatty marrow is replaced by a fully pneumatized sinus lined by respiratory epithelium [[4], [5], [6]].

In rare cases for no specific reason, the pneumatization fails before the respiratory mucosa fully extends into the site of fatty marrow conversion. In this case, atypical fatty marrow remains permanently around the sphenoid sinus even in adult hood (imitating a lesion), thus called arrested pneumatisation [7].

Arrested pneumatization is usually mistaken for a tumour because the sphenoid bone is associated with several developmental diseases.**Individuals with arrested pneumatization of the skull base are usually asymptomatic and the variant is most often discovered incidentally when imaging is performed for a separate cause.**

The aim of this study was to highlight the imaging features of arrested pneumatization of the skull base to aid the clinician in recognizing this developmental variant which can be that can be confused with serious diseases of the skull base (tumours) and be symptomatic.

Our work consists of a single case report and has been reported in line with the SCARE 2020 criteria [8].

## 2 Case presentation

The patient was 15 years old male, with no known pathological history or toxic habits. He consulted an Otorhinolaryngologist for chronic headaches and left-sided hearing loss. Physical examination was normal. Otoscopy was normal revealing a normal eardrum bilaterally. The tonal audiogram revealed a moderate sensorineural hearing loss of 46 dB in the left ear.

**Fig. 1:**
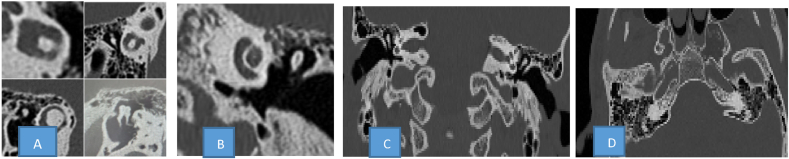
CT-scan of petrous bone was normal: labyrinths, semicirculars canals, the ossicles of middle ear, tympanic cavity, inner ear structures are normal. Hyper-pneumatazetion of mastoid cells and lesion of left sphenoid bone. Presence of a hypodense lesion in the left sphenoid bone.

Petrous bone CT scan was performed without any particular abnormality in the middle ear: the tympanic membrane and the ossicular chain are normal. However, a hypodense lesion in the sphenoid bone was noted. The initial diagnosis was a lipoma (Fig. 1).

**Fig. 2:**
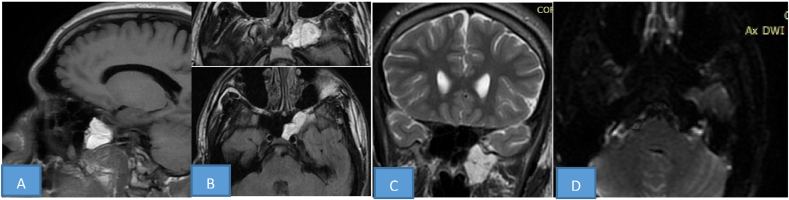
Brain RM image T1, T2 and DWI**:** Sagittal (A) and axial (B) T1 weighted (W) SE, and coronal(C) T2 axial (D) DWI: MR images show well defined lobulated non-expansive lesion located at the left sphenoid sinus pneumatization site (arrows). The lesion demonstrates increased signal on both T1W and T2W images which is consistent with fat signal.

To better characterize the lesion radiologist then requested a brain MRI. The images show a well-defined, non-expanding lobulated lesion located at the site of pneumatisation of the left sphenoid sinus (arrows). The lesion shows increased signal on T1W and T2W images, which is consistent with the fat signal. The second diagnosis was nasosinus fibrous dysplasia (Fig. 2).

**Fig. 3:**
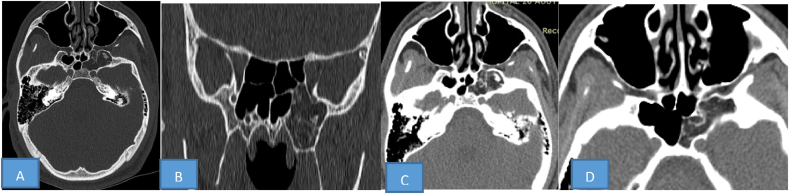
Brain CT-Scan: revealed: absence of aeration of the left sphenoidal sinus. Non-eroded, non-expansile lesion of fatty density lesion with a well-demarcated and internal curvilinear calcification in the location of left sphenoid sinus. The margin was osteosclerotic, and the adjacent bony structure was intact and showed no mass effect.

In view the diagnoses discrepancy, the patient was referred to our department for a specialist opinion. An additional brain scan revealed. We completed the examination with a brain scan in order to explore the whole bone structure. The images show: a non-eroded, non-expansive fatty density lesion with a well-defined internal curvilinear calcification opposite the left sphenoid sinus. The lesion extended into the sphenopalatine cleft and pterygoid process, its margin was osteosclerotic, and the adjacent bone structure was intact and showed no mass effect (Fig. 3). Our final diagnosis was an arrested pneumatisation.

Since this is a normal development variant, no additional examination or treatment was performed.

In our case no other cause is found to explain his symptomatology. A clinical-radiological follow-up was instituted to ensure the benignity of the lesion.

After one year of follow-up no aggravation of symptoms or complications were observed. The first radiological control showed a stable aspect of the lesion.

## 3 Discussion

During normal development, a process known as red-to yellow marrow conversion takes place, in which the adipose tissue within the red marrow increases in relative percentage. Several studies indicate that pneumatization of the cranial bones, such as the paranasal sinuses and mastoid air cells, only occurs after red-to-yellow conversion. If the conversion process does not begin or is not completed, the pneumatization process is arrested and the area that is normally aerated is instead filled with yellow marrow [4,7,9], hence termed arrested pneumatisation. A pseudo-tumour lesion is found on the site.

Arrested pneumatization of the skull base is not well recognized by many clinicians and radiologists and can often be confused with other pathologies such as fibrous dysplasia, chordoma, chondrosarcoma, intraosseous lipoma, intraosseous hemangioma, hamartoma, ossifying fibroma, enchondroma, and fibrous osteoma [4,[7], [8], [9], [10], [11], [12]].

Unless complicated, Individuals with arrested pneumatization of the skull base are usually asymptomatic and the variant is most often discovered incidentally when imaging is performed for a separate cause. However in such cases of arrested pneumatization of sinus sphenoid, symptoms occurs mimicking serious disorders [12].

Our patient is one of the rare cases of arrested pneumatization revealed by non-specific symptoms such as headache and hearing loss.

Imaging perspective, a set of imaging criteria (Welker's criteria) have been proposed to establish the diagnosis: the lesion must be located at a site of normal pneumatisation or a site of recognized accessory pneumatisation; the lesion must be non-expansive; the lesion should have sclerotic, well-circumscribed margins; the lesion should show fatty content; on CT, internal curvilinear calcifications should be present - these should be morphologically different from the “ring and arc” type calcifications found with chondroid series tumours; any associated skull base foramina should retain their normal appearance [6,7,9,12,13].

Our case fulfills all radiologics criterias of an arrested pneumatisation of sphenoid sinus. In some cases the full range of radiological features are not found requiring regular follow-up.

## 4 Conclusion

Arrested pneumatization of the skull base is an anatomical variant that most commonly occurs in the sphenoid sinus. The diagnosis is made when the lesion is located in a site of normal pneumatization, especially the sinuses. If the lesion characteristics are complete the diagnosis of arrested pneumatization can be made with confidence, eliminating the need for additional interventions such as biopsy or surgery. Occasionally, the lesion may not fulfil all these radiological criteria, in which case serial imaging surveillance is required to establish benignity.

## Ethical approval

Written informed consent was obtained from the patient for publication of this case report and accompanying images. A copy of the written consent is available for review by the Editor-in-Chief of this journal on request.

## Sources of funding for your research

The authors declared that this study has received no financial support.

## Author contribution

**Harouna** Maman Siradji: Corresponding author, writing the paper.
Fadoul Achta: writing the paper.
Aghrib Fatiha: writing the paper.
Boussa Tressia: writing the paper.
Belgadir Hasna: Correction of the paper.
Elbenna Naima: Correction of the paper.

## Consent

Written informed consent was obtained from the patient for publication of this case report and accompanying images. A copy of the written consent is available for review by the Editor-in-Chief of this journal on request.

## Registration of research studies

1. Name of the registry: researchregistry

2. Unique Identifying number or registration ID: 7110.

3. Hyperlink to your specific registration (must be publicly accessible and will be checked):

## Guarantor

HAROUNA DJATAOU MAMAN SIRADJI.

## Declaration of competing interest

Authors of this article have no conflict or competing interests. All of the authors approved the final version of the manuscript.
